# Research on the game of manufacturing capacity sharing based on prospect theory

**DOI:** 10.1038/s41598-023-45189-x

**Published:** 2023-10-23

**Authors:** Tian-Yu Wang, Hao Zhang

**Affiliations:** School of Economics and Management, Jiangsu University of Science and Technology, Zhenjiang, 212100 China

**Keywords:** Evolutionary theory, Engineering

## Abstract

In order to investigate the strategy choice of each player in capacity sharing, the article constructs a tripartite game model based on capacity provider-capacity demander-government, introduces the prospect theory and conducts numerical simulation analysis using MATLAB. The results show that capacity sharing in the manufacturing industry is related to three parties: capacity providers, capacity demanders and the government, and their strategies in the game process influence each other; the sensitivity of capacity providers and capacity demanders is higher than that of the government; the increase of risk-return coefficient and loss-avoidance coefficient is conducive to the evolution of subjects to the ideal state.

## Introduction

The deep integration of “sharing economy + Internet” has promoted the transformation^1–3^ and upgrading of the manufacturing industry^4, 5^, and the sharing of manufacturing capacity is an effective way to alleviate the imbalance between the supply and demand of capacity, and has become a new development in the manufacturing industry^6^, which is conducive to improving the utilization rate of redundant resources and is of great significance to the high-quality development of the manufacturing industry^7–9^. The process of manufacturing capacity sharing can lead to unreasonable capacity matching and increased sunk costs due to the default of the other party, thus requiring government regulation. It is of practical significance for the good development of the manufacturing industry to study how to promote the capacity-sharing behavior among manufacturing industries so as to maximize the interests of all parties.

Research has been conducted on the sharing of manufacturing capacity. Based on evolutionary game theory, Zhang et al. has built a blockchain incentive model for both sides and designed smart contracts to encourage companies to participate in “bookkeeping” and enhance the stability of blockchain shared manufacturing^10^. Chen et al. constructed the evolutionary game of capacity sharing between enterprises and enterprises and the game between enterprises and government around green and low carbon innovation, and analyzed the evolutionary process of enterprises’ strategy choice based on the model^11^. Zhao et al. constructed an evolutionary game model of platform-capacity idlers and capacity demanders, and explored the impact of transaction costs and cost effects of corporate low-carbon capacity on system stability^12^. To understand the behavioral decisions of manufacturing firms in the context of shared manufacturing, Lesser et al. used evolutionary game theory to construct a model and MATLAB to analyze the factors influencing capacity sharing^13, 14^. TIAN Chen coupled and analyzed blockchain and manufacturing capacity sharing, and constructed a blockchain-based manufacturing capacity sharing model to help the transformation and upgrading of traditional manufacturing enterprises^15^.

However, in the actual manufacturing capacity sharing process, each subject’s strategy choice will depend on its perception of the value of the costs and benefits in the manufacturing capacity sharing process. Although the traditional game studies compensate for the limitations of the expected utility theory, they still lack the study of the subjective consciousness of the subjects^16, 17^. Based on prospect theory and evolutionary game theory, Shen et al. constructed a game model between local governments and polluting enterprises, and studied the factors influencing decision-making behavior using simulation techniques^18^. Dong et al. constructed a tripartite evolutionary game model of regulators, electricity retailers and consumers from a finite rationality perspective to explore the impact of dynamic pricing on electricity promotion. The results of the study indicate that agency subsidies, promotion costs, and psychological factors of consumers all have an impact^19^. The issue of carbon regulation has attracted attention with the low-carbon economy. Sun et al. constructed an evolutionary game model under the prospect theory to analyze the stabilization strategies of government and enterprises under different air risks, and the results of the study found that the strategy choices under different air risks are different^20^.

In summary, the correlation for manufacturing capacity sharing is mostly based on two subjects making decisions, and most of them are only limited to evolutionary game theory, while ignoring the perceptual differences between people in the decision-making process^21^. What are the strategic choices of each party involved in the sharing of manufacturing capacity? What is the sensitivity of each player to risk and loss? Based on this, the article constructs a tripartite game study among capacity providers, capacity demanders and government to explore the stable strategies of the model; at the same time, prospect theory is introduced to analyse the changes of each subject’s sensitivity to risks and losses^22^; finally, the article summarizes the results and proposes relevant countermeasures.

## Problem analysis and model building

### Problem analysis

**Figure 1:**
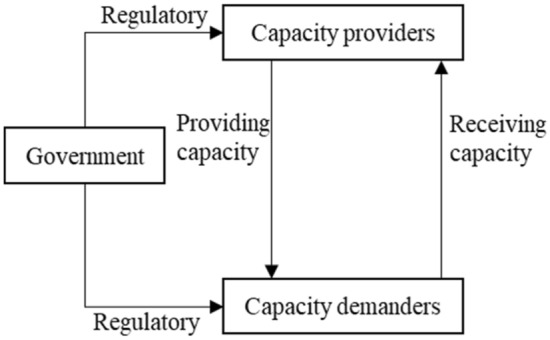
Relationship between capacity providers, capacity demanders and government.

The process of manufacturing capacity sharing involves multiple entities, including capacity providers, capacity demanders and the government. The capacity provider can choose to provide capacity to improve the utilization rate of redundant resources; the capacity demander can choose to receive capacity to expand the production capacity of the enterprise^23^. If there is only the manufacturing capacity provider and the demand side, there may be fraud, default, and false sharing for benefits, so the government needs to regulate. Government regulation plays an important role in the process of manufacturing capacity sharing, which can not only effectively stimulate the sharing behavior of both sides, promote the utilization of idle resources, drive income, but also regulate the standard, good and sustainable development of manufacturing industry. If the capacity provider does not provide capacity, the capacity demander does not receive capacity, and the government does not regulate, this will result in wasted manufacturing resources, reduced government credibility, and stagnant development of the manufacturing industry. The tripartite main parties repeatedly play games based on their own benefits and costs. The relationship between the three is shown in Fig. 1.

### Model assumptions and construction

In view of the above analysis, the model assumptions are as follows:

#### Hypothesis 1

In the process of manufacturing capacity sharing, there are tripartite parties, the capacity provider, the capacity demander and the government, and all three parties base their decisions on their own maximum benefits. The strategy choice space of the capacity provider is {share, don’t share}, and the probability of choosing “share” is $x$ and the probability of choosing “don’t share” is 1 − $x$; The strategy choice space for the demand side is {receive, don’t receive}, with a probability of $y$ choosing “receive” and 1 − $y$ choosing “don’t receive”; The government’s strategy choice space is {regulate, don’t regulate}, and the probability of choosing “regulate” is $z$, and the probability of choosing “don’t regulate” is The probability of choosing “not to regulate” is 1 − $z$.

#### Hypothesis 2

The base benefit to the government is $R_{1}$, when the government chooses to regulate, the cost of regulation in terms of manpower, time and management is $C_{1}$. When the capacity provider shares the capacity and the capacity demander receives the capacity, the government regulation gains additional gains in terms of credibility enhancement and trust is s. Conversely, when the government does not regulate, the loss of external condemnation is $R_{o}$. When the capacity provider cooperates with the capacity demander, the additional loss is $S_{1}$.

#### Hypothesis 3

The base benefit to the capacity provider is $R_{2}$. When the capacity provider chooses to share capacity, it incurs costs of manpower, time, management $C_{2}$; if the capacity provider shares capacity but the capacity demander does not receive capacity, the provider gains a digital level of benefit $R_{a}$, even though the cooperation is not reached; when the capacity provider chooses not to share capacity, it suffers a loss of trust $S_{2}$. If the capacity provider shares capacity and the capacity demander receives capacity, both parties receive a synergy benefit factor $\theta$, quantifying the level of sharing as $Q$^24^.

#### Hypothesis 4

The base benefit for the capacity demander is $R_{3}$, and the cost when choosing to receive capacity is $C_{3}$. If the capacity demander receives capacity, but the capacity provider does not share capacity, it receives a digital level benefit of $R_{b}$; the capacity demander suffers a loss of trust of $S_{3}$ when it does not receive capacity.

Based on the above assumptions, a benefit matrix is constructed as shown in Table 1.

**Table 1:** Game payoff matrix.

	Capacity provider sharing $x$		Capacity provider not sharing $1 - x$	
	Capacity demander receiving $y$	Capacity demander not receiving $1 - y$	Capacity demander receiving $y$	Capacity demander not receiving $1 - y$
Government regulation $z$	$R_{2} - C_{2} + \theta Q + R_{a}$	$R_{2} - C_{2} + R_{a}$	$R_{2} - S_{2}$	$R_{2}$
	$R_{3} - C_{3} + \theta Q + R_{b}$	$R_{3} - S_{3}$	$R_{3} - C_{3} + R_{b}$	$R_{3}$
	$R_{1} - C_{1} + R_{o}$	$R_{1} - C_{1}$	$R_{1} - C_{1}$	$0$
Government not regulation $1 - z$	$R_{2} - C_{2} + \theta Q + R_{a}$	$R_{2} - C_{2} + R_{a}$	$R_{2} - S_{2}$	$R_{2}$
	$R_{3} - C_{3} + \theta Q + R_{b}$	$R_{3} - S_{3}$	$R_{3} - C_{3} + R_{b}$	$R_{3}$
	$R_{1} - S_{1} - S_{o}$	$R_{1} - S_{1}$	$R_{1} - S_{1}$	$0$

### Evolutionary strategy analysis

The evolutionary strategy and evolutionary path of the capacity provider, the capacity demander and the government can be obtained according to the benefit matrix.

#### Analysis of the evolutionary of production capacity provider strategies

$E_{a1}$ denotes the benefit if the capacity provider chooses to “share”, $E_{a2}$ denotes the benefit if the capacity provider chooses not to “share”, and $E_{a}$ is the average expected benefit for the capacity provider, then:

1 $$\begin{aligned} E_{a1} = & yz(R_{2} - C_{2} + \theta Q + R_{a} ) + y(1 - z)(R_{2} - C_{2} + \theta Q + R_{a} ) \\ & + {\kern 1pt} z(1 - y)(R_{2} - C_{2} + R_{a} ) + (1 - y)(1 - z)(R_{2} - C_{2} + R_{a} ) \\ = & y\theta Q + R_{2} - C_{2} + R_{a} , \\ \end{aligned}$$

2 $$\begin{aligned} E_{a2} = & yz(R_{2} - S_{2} ) + y(1 - z)(R_{2} - S_{2} ) \\ {\kern 1pt} & + {\kern 1pt} z(1 - y)R_{2} + (1 - y)(1 - z)R_{2} \\ {\kern 1pt} = & R_{2} - yS_{2} , \\ \end{aligned}$$

3 $$E_{a} = xE_{a1} + (1 - x)E_{a2} .$$

The replication dynamic equation for the capacity provider is:

4 $$\begin{aligned} F(x) = & \frac{dx}{{dt}} = x(Ea - E_{a1}) \\ {\kern 1pt} = & x(1 - x)(E_{a1} - E_{a2}) \\ {\kern 1pt} = & x(1 - x)[y\theta Q - C_{2} + R_{a} + y S_{2} ]. \\ \end{aligned}$$

This point is the point of evolutionary stability when the replication dynamic equation is 0^25^. Let $F(x)$ = 0, then $y*$ = $C_{2}$ − $R_{a}$/$\theta Q$ + $S_{2}$, at which point the strategy choice of the capacity provider is in a stable state. When $y$ ≠ $y*$, then $x$ = 0 and $x$ = 1 are stable points, to be discussed in separate cases:

When $y$ > $y*$, $F^{\prime}x(0)$ > 0, $F^{\prime}x(1)$ < 0, then $x$ = 1 is the ESS, the capacity provider tends to choose the “sharing” strategy; when $y$ < $y*$, $F^{\prime}x(0)$ < 0, $F^{\prime}x(1)$ > 0, then $x$ = 0 is the ESS, the capacity provider tends to choose the “non-sharing” strategy.

**Figure 2:**
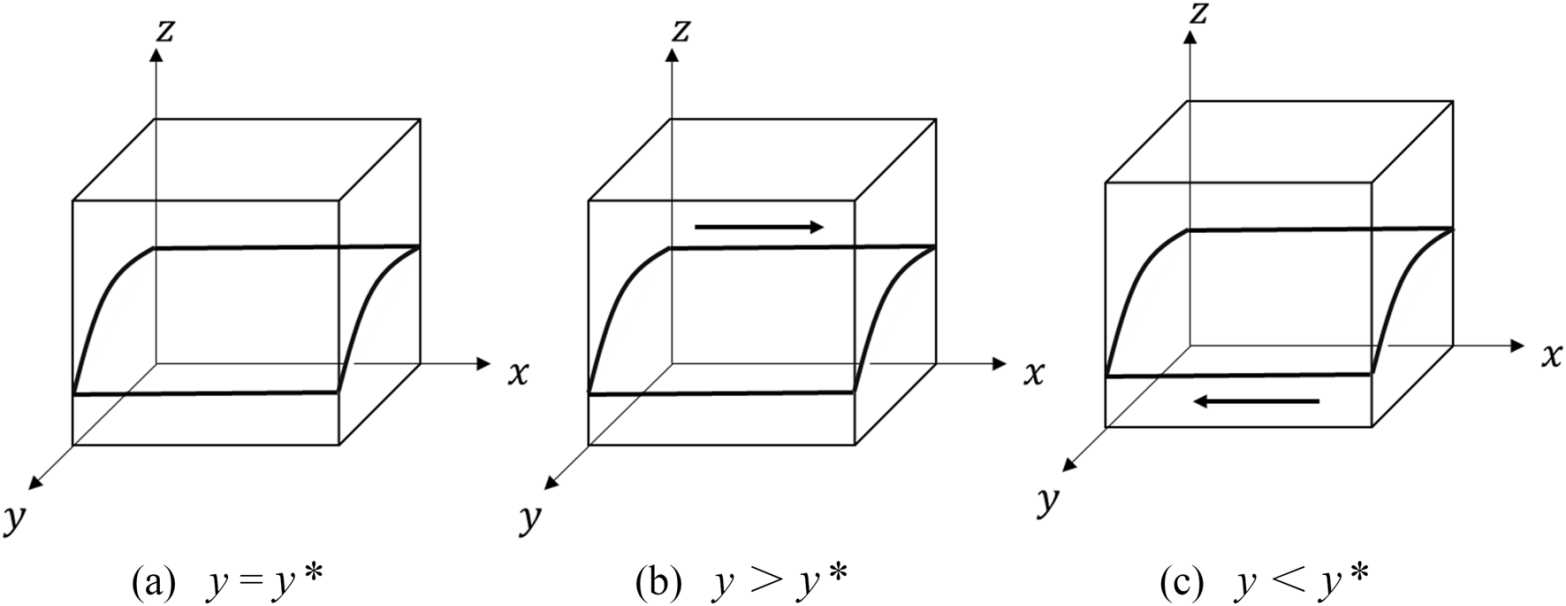
Evolution of the strategy choice on the provider side of capacity.

The evolutionary phase diagram of the capacity provider’s strategy choice is shown in Fig. 2.

### Analysis of the evolutionary of production capacity demander strategies

$E_{b1}$ denotes the revenue if the capacity-demanding party chooses to “receive”, $E_{b2}$ denotes the revenue if the capacity-demanding party chooses not to “receive”, and $E_{b}$ is the average expected revenue of the capacity-demanding party, then:

5 $$\begin{aligned} Eb1 = & xz(R_{3} - C_{3} + \theta Q + R_{b} ) + x(1 - z)(R_{3} - C_{3} + \theta Q + R_{b} ) \\ & + {\kern 1pt} z(1 - x)(R_{3} - C_{3} + R_{b} ) + (1 - x)(1 - z)(R_{3} - C_{3} + R_{b} ) \\ = & x\theta Q + R_{3} - C_{3} + R_{b} , \\ \end{aligned}$$

6 $$\begin{aligned} Eb2 = & xz(R_{3} - S_{3} ) + x(1 - z)(R_{3} - S_{3} ) \\ & + {\kern 1pt} z(1 - x)R_{3} + (1 - x)(1 - z)R_{3} \\ {\kern 1pt} = & R_{3} - xS_{3} , \\ \end{aligned}$$

7 $$E_{b} = yE_{b1} + (1 - y)E_{b2} .$$

The replication dynamic equation for the capacity demander is:

8 $$\begin{aligned} F(y) = & \frac{dy}{{dt}} = y(E_{b} - E_{b1} ) \\ = & y(1 - y)(E_{b1} - E_{b2} ) \\ = & y(1 - y)[x\theta Q - C_{3} + R_{b} + xS_{3} ]. \\ \end{aligned}$$

Similarly, if $F(y)$ = 0, then $x*$ = $C_{3}$ − $R_{b}$/$\theta Q$ + $S_{3}$, at which point the strategy choice on the demand side of capacity is in a steady state. When $x$ ≠ $x*$, then $y$ = 0 and $y$ = 1 are stable points, to be discussed in separate cases:

When $x$ > $x*$, $F^{\prime}y_{(0)}$ > 0, $F^{\prime}y_{(1)}$ < 0, then $y$ = 1 is the ESS, i.e. the capacity demander tends to choose the “receive” strategy; when $x$ < $x*$, $F^{\prime}y_{(0)}$ < 0, $F^{\prime}y_{(1)}$ > 0, then $y$ = 0 is the ESS, the capacity demander tends to choose the “do not receive” strategy.

**Figure 3:**
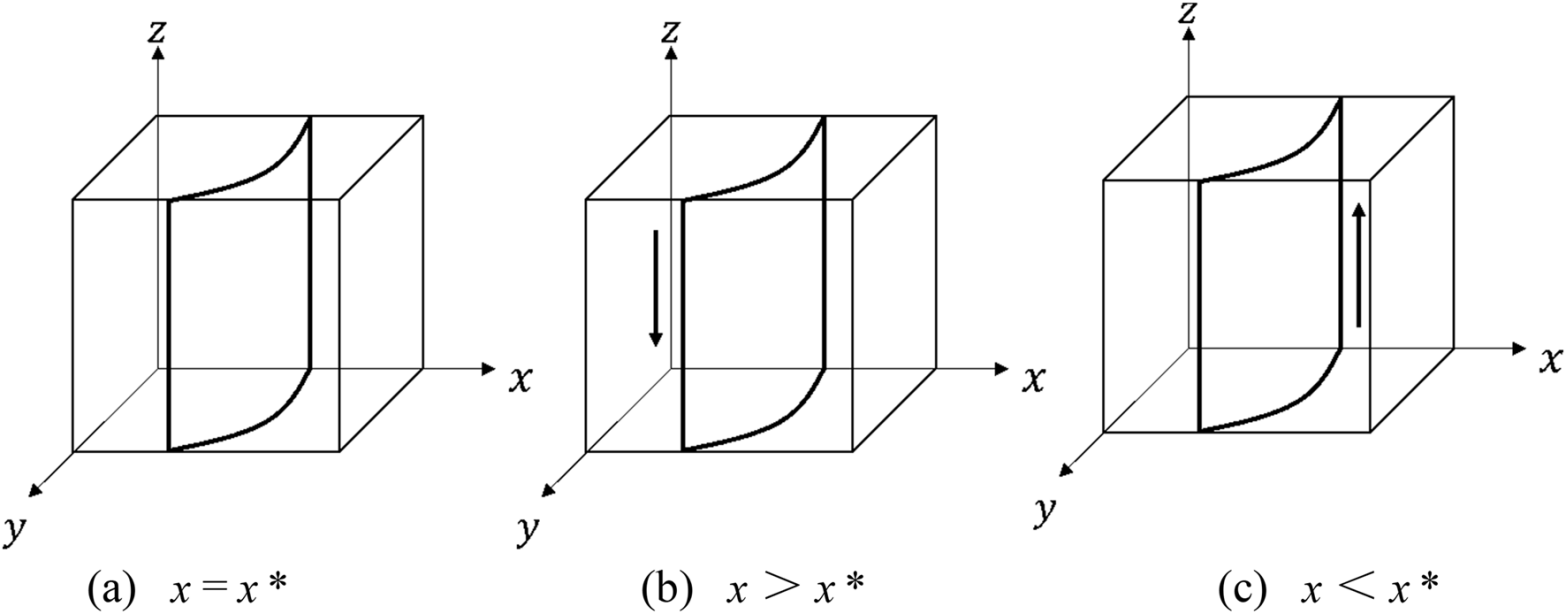
Evolution of the strategy choice on the demander side of capacity.

The evolutionary phase diagram of the strategy choice of the capacity demander is shown in Fig. 3.

### Analysis of the evolutionary of government strategies

$Eu1$ denotes the return if the government chooses to “regulate”, $E_{u2}$ denotes the return if the government chooses not to “regulate”, and $E_{u}$ is the average expected return to the government, then:

9 $$\begin{aligned} Eu1 = & xy(R_{1} - C_{1} + R_{o} ) + x(1 - y)(R_{1} - C_{1} ) + {\kern 1pt} y(1 - x)(R_{1} - C_{1} ) \\ {\kern 1pt} = & xy(R_{o} - R_{1} + C_{1} ) + (x + y)(R_{1} - C_{1} ), \\ \end{aligned}$$

10 $$\begin{aligned} Eu2 = & xy(R_{1} - S_{1} - So) + x(1 - y)(R_{1} - S_{1} ) + {\kern 1pt} y(1 - x)(R_{1} - S_{1} ) \\ {\kern 1pt} = & xy(S_{1} - S_{o} - R_{1} ) + (x + y)(R_{1} - S_{1} ), \\ \end{aligned}$$

11 $$E_{u} = zE_{u1} + (1 - z)E_{u2} .$$

The replication dynamic equation for the government is:

12 $$\begin{aligned} F(z) = & \frac{dz}{{dt}} = z(E_{u} - E_{u1} ) \\ {\kern 1pt} = & z(1 - z)(E_{u1} - E_{u2} ) \\ {\kern 1pt} = & z(1 - z)[xy(R_{o} + C_{1} - S_{1} + S_{o} ) + (x + y)(S_{1} - C_{1} )]. \\ \end{aligned}$$

Similarly, let $F(z)$ = 0, then $y*$ =  − $x$($S_{1}$ − $C_{1}$)/$x$($R_{o}$ + $C_{1}$ − $S_{1}$ + $S_{o}$), when the government’s strategy choice is in a steady state. When $y$ ≠ $y*$, then $z$ = 0 and $z$ = 1 are stable points, to be discussed in separate cases.

When $y$ > $y*$, $F^{\prime}z_{(0)}$ > 0 and $F^{\prime}z_{(1)}$ < 0, then $z$ = 1 is the ESS, i.e. the government tends to choose a “regulatory” strategy; when $y$ < $y*$, $F^{\prime}z_{(0)}$ < 0 and $F^{\prime}z_{(1)}$ > 0, then $z$ = 1 is the ESS, the government tends to choose a “non-regulatory” strategy.

**Figure 4:**
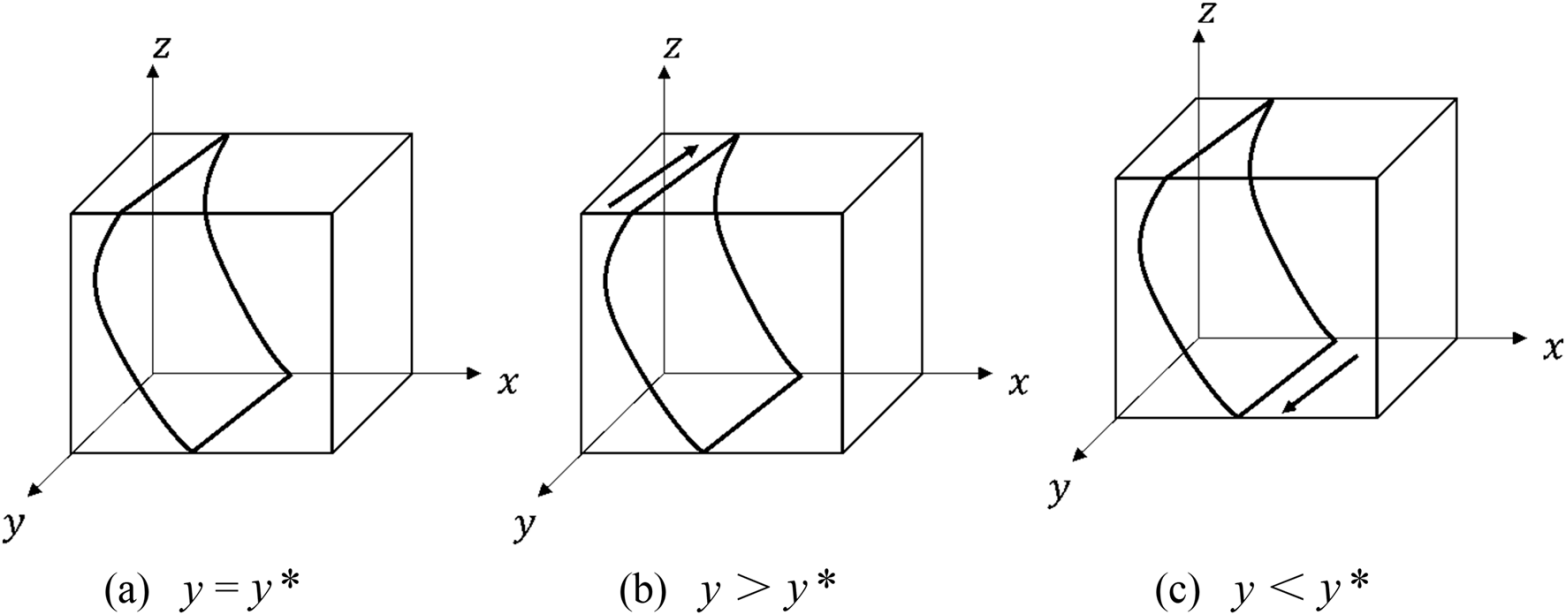
Evolution of the government’s choice of strategy.

The evolutionary phase diagram of the government’s strategy choice is shown in Fig. 4.

### System stability strategy analysis

According to the method proposed by Friedman^26^, the Jacobian matrix is applied to determine the stability of the system in. the Jacobian matrix is:

13 $$\begin{aligned} J = & \left[ {\begin{array}{*{20}c} {\frac{\partial F(x)}{{\partial x}}} & {\frac{\partial F(x)}{{\partial y}}} & {\frac{\partial F(x)}{{\partial z}}} \\ {\frac{\partial F(y)}{{\partial x}}} & {\frac{\partial F(y)}{{\partial y}}} & {\frac{\partial F(y)}{{\partial z}}} \\ {\frac{\partial F(z)}{{\partial x}}} & {\frac{\partial F(z)}{{\partial y}}} & {\frac{\partial F(z)}{{\partial z}}} \\ \end{array} } \right] \\ \\ {\kern 1pt} = & \left[ {\begin{array}{*{20}l} {(1 - 2x)[y\theta Q - C_{2} + R_{a} + y S_{2} ]} \hfill & {x(1 - x)(\theta Q + S_{2} )} \hfill & 0 \hfill \\ {y(1 - y)(\theta Q + S3)} \hfill & {(1 - 2y)[x\theta Q - C_{3} + R_{b} + xS3} \hfill & 0 \hfill \\ \begin{gathered} z(1 - z)[y(Ro + C1 - S1 \hfill \\ + So) + S1 - C1)] \hfill \\ \end{gathered} \hfill & \begin{gathered} z(1 - z)[x(Ro + C1 - S1 \hfill \\ + So) + S1 - C1)]] \hfill \\ \end{gathered} \hfill & \begin{gathered} (1 - 2z)[xy(Ro + C1 - S1 \hfill \\ + So) + (x + y)(S1 - C1)] \hfill \\ \end{gathered} \hfill \\ \end{array} } \right]. \\ \end{aligned}$$

Let $F(x)$ = $F(y)$ = $F(z)$ = 0, resulting in equilibrium and saddle points: (0,0,0), (0,1,0), (0,0,1,), (0,1,1), (1,0,0), (1,0,1), (1,1,0), (1,1,0), (1,1,1), (1,1,1), ($x*$, $y*$, $z*$). In the process of manufacturing capacity sharing, capacity providers share out redundant resources, capacity demanders are willing to receive capacity, and, at the same time, government regulation to avoid default and fraud has good implications for the quality development of manufacturing. When capacity providers share capacity, capacity demanders receive capacity, and the government regulates, when in the evolutionary state (1,1,1), the ideal state is reached and benefits are maximized. Therefore, the article carries out the analysis of evolutionary conditions and the study of pathways in the state of (1,1,1). Bringing (1,1,1) into the Jacobian matrix yields:

14 $$J = \left[ {\begin{array}{lll} { - (\theta Q - C_{2} + R_{a} + S_{2} )} & 0 & 0 \\ 0 & { - (\theta Q - C_{3} + R_{b} + S_{3} )} & 0 \\ 0 & 0 & { - (R_{o} - C_{1} - S_{1} + S_{o} )} \\ \end{array} } \right].$$

According to Lyapunov’s first method, the steady state of the system is reached at this point when the eigenvalues are non-negative real numbers, otherwise it is a non-stable transitive state^27^. To be stable in the (1,1,1) state, − ($\theta Q$ − $C_{2}$ + $R_{a}$ + $S_{2}$) < 0, − ($\theta Q$ − $C_{3}$ + $R_{b}$ + $S_{3}$) < 0, − ($R_{o}$ − $C_{1}$ − $S_{1}$ + $S_{o}$) < 0, the expected benefits of government regulation, with capacity providers choosing to ‘share’ and capacity demanders choosing to ‘receive’ The expected benefits outweigh the expected costs and losses.

## Model optimization under prospect theory condition

The previous section demonstrated the strategic choices made by the various agents in the manufacturing capacity sharing process, which are made based on the values of costs and benefits. However, in the actual sharing process, the actors also take into account other factors, for example, although the capacity provider, the capacity demander, or the government makes a decision that is beneficial to them, they may also consider the riskiness of the external environment and end up choosing the opposite strategy. All of these take into account the perceived value. So, although evolutionary games break through the limitations of the traditional theory of expected utility to compensate for the game process, the process by which subjects make decisions still lacks a subjective level of consciousness. Based on this, Kahneman in 1979 proposed a prospect theory that is more in line with realistic decision making from a psychological perspective^28^. Prospect theory holds that the subject is risk averse when facing profit, that is, he is inclined to choose the certain profit, rather than the risky choice that may obtain greater profit. In the face of loss is risk preference, that is, the tendency to choose the option that is likely to cause a greater loss, rather than a certain loss. Each agent will make a reference point in mind, and then measure each result above or below this reference point, above the reference point is a profit, below the reference point is a loss. Prospect value $V$ consists of a weight function $\pi (P_{i} )$ and a value function $U(\Delta \omega_{i} )$, which can be expressed as:

15 $$V = \mathop \sum \limits_{i = 1} \pi (P_{i} )U(\Delta \omega_{i} ),$$

16 $$U\left( \omega \right) = \left\{ {\begin{array}{*{20}l} {\omega^{\alpha } \left( {\omega > 0} \right)} \hfill \\ { - \lambda \left( { - \omega } \right)^{\alpha } \left( {\omega < 0} \right)} \hfill \\ \end{array} } \right.,$$

17 $$\pi \left( {P_{i} } \right) = \frac{{p^{r} }}{{\left( {p^{r} + \left( {1 - P} \right)^{\gamma } } \right)^{\frac{1}{r}} }}.$$

$\pi (P_{i} )$ represents the probability of event $i$ occurring, $\Delta \omega_{i}$ represents the decision weight of the subject measuring event $i$, $\Delta \omega$ represents the difference between the subject’s actual payoff before and after the game, $\alpha$ (0 < $\alpha$ < 1) represents the marginal decreasing value function of the decision maker’s perceived gains and losses, with larger values of $\alpha$ indicating a greater preference for risk-taking. $\lambda$ ($\lambda$ > 1) represents the loss aversion coefficient, with larger values of $\lambda$ indicating a higher sensitivity to loss.

According to the prospect theory, the risk sensitivity coefficients of the capacity provider, the capacity demander and the government are set as $\alpha_{1}$, $\alpha_{2}$, $\alpha_{3}$, and the loss avoidance coefficients as $\lambda_{1}$, $\lambda_{2}$, $\lambda_{3}$. For the uncertain gains and losses:$R_{o}$, $R_{a}$, $R_{b}$, $S_{o}$, $S_{2}$, $S_{3}$.

Substituted into $F(x)$, $F(y)$ and $F(z)$ respectively, the replication dynamics equations are obtained as follows:

18 $$F(x) = x(1 - x)[y\theta Q - C_{2} + x\mathop {R_{a} }\nolimits^{\alpha 1} + y(1 - x)\lambda_{1} \mathop {S_{2} }\nolimits^{\alpha 1} ],$$

19 $$F(y) = y(1 - y)[x\theta Q - C_{3} + y\mathop {R_{b} }\nolimits^{\alpha 2} {\kern 1pt} {\kern 1pt} + x(1 - y)\lambda_{2} \mathop {S_{3} }\nolimits^{\alpha 2} ],$$

20 $$F(z) = z(1 - z)[xy(z\mathop {R_{o} }\nolimits^{\alpha 3} + C_{1} - S_{1} {\kern 1pt} {\kern 1pt} + (1 - z)\lambda_{3} \mathop {S_{o} }\nolimits^{\alpha 3} ] + (x + y)(S_{1} - C_{1} )].$$

## MATLAB simulation analysis

**Figure 5:**
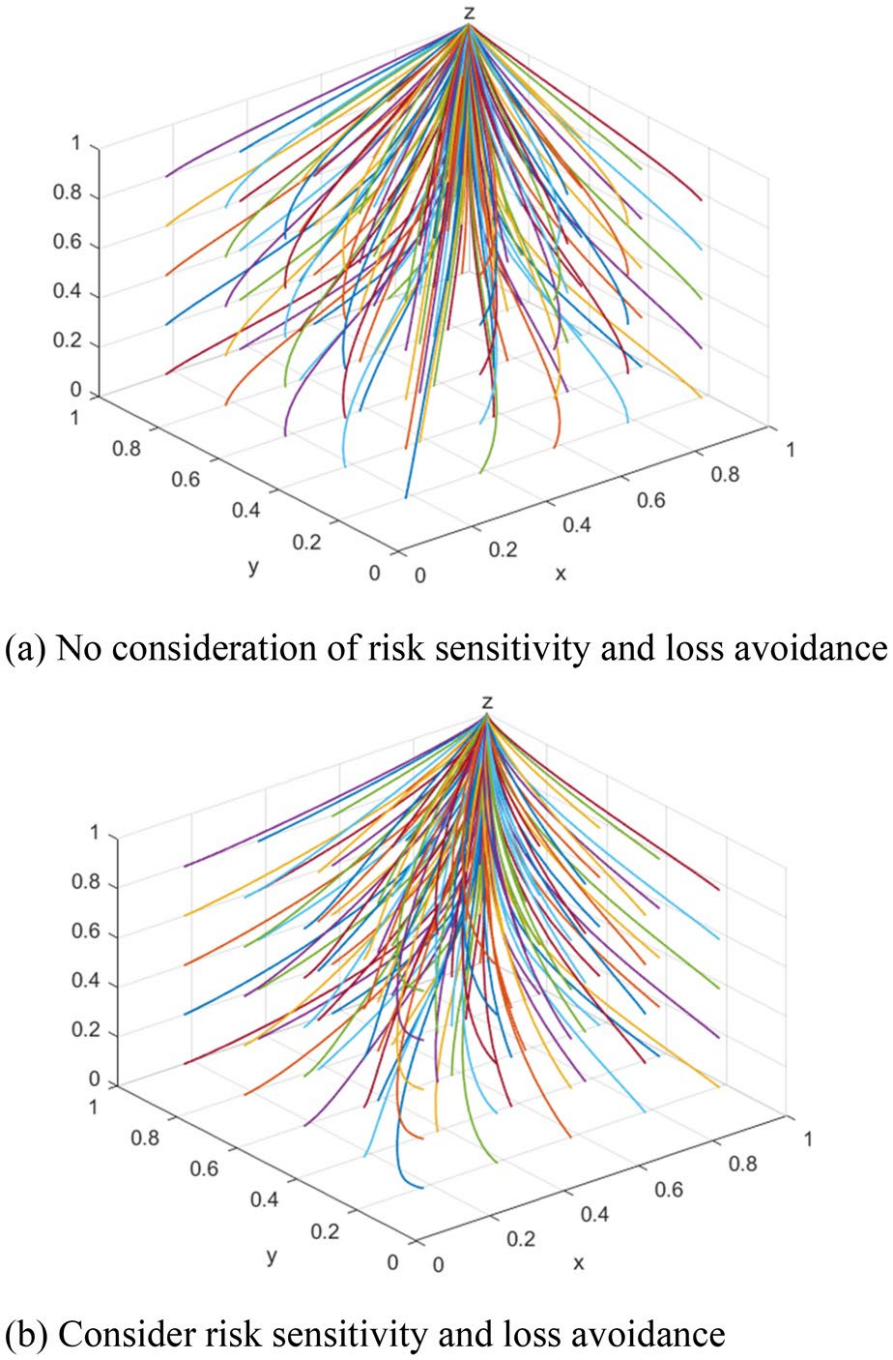
Evolution in different contexts.

For further analysis, the article assigns values to the parameters in conjunction with the stability conditions of (1,1,1). Assuming $\theta$ = 0.8, $Q$ = 20,$C_{1}$ = 8, $C_{2}$ = 4, $C_{3}$ = 2, $R_{o}$ = 13, $R_{a}$ = 10, $R_{b}$ = 8, $S_{o}$ = 10, $S_{1}$ = 8, $S_{2}$ = 5, $S_{3}$ = 7, the decision maker’s preferences can be roughly expressed when the risk sensitivity coefficient $\alpha_{1}$ = $\alpha_{2}$ = $\alpha_{3}$ = 0.88 and the loss aversion coefficient $\lambda_{1}$ = $\lambda_{2}$ = $\lambda_{3}$ = 2.25 according to Tversky A^29^. The evolutionary results are shown in Fig. 5.

As can be seen from Fig. 5a, when the limited rationality of the tripartite subject is not considered, the system will converge to the optimal state (1,1,1), and when under the prospect theory condition, as can be seen from Fig. 5b, the system will still eventually converge to (1,1,1). Although the results of the model before and after optimization both evolved to (1,1,1), the evolution process was different, indicating that there were factors in the optimized model that affected the judgment of each subject and made the preference strategy choice. Therefore, the model of introducing foreground theory is worth studying.

**Figure 6:**
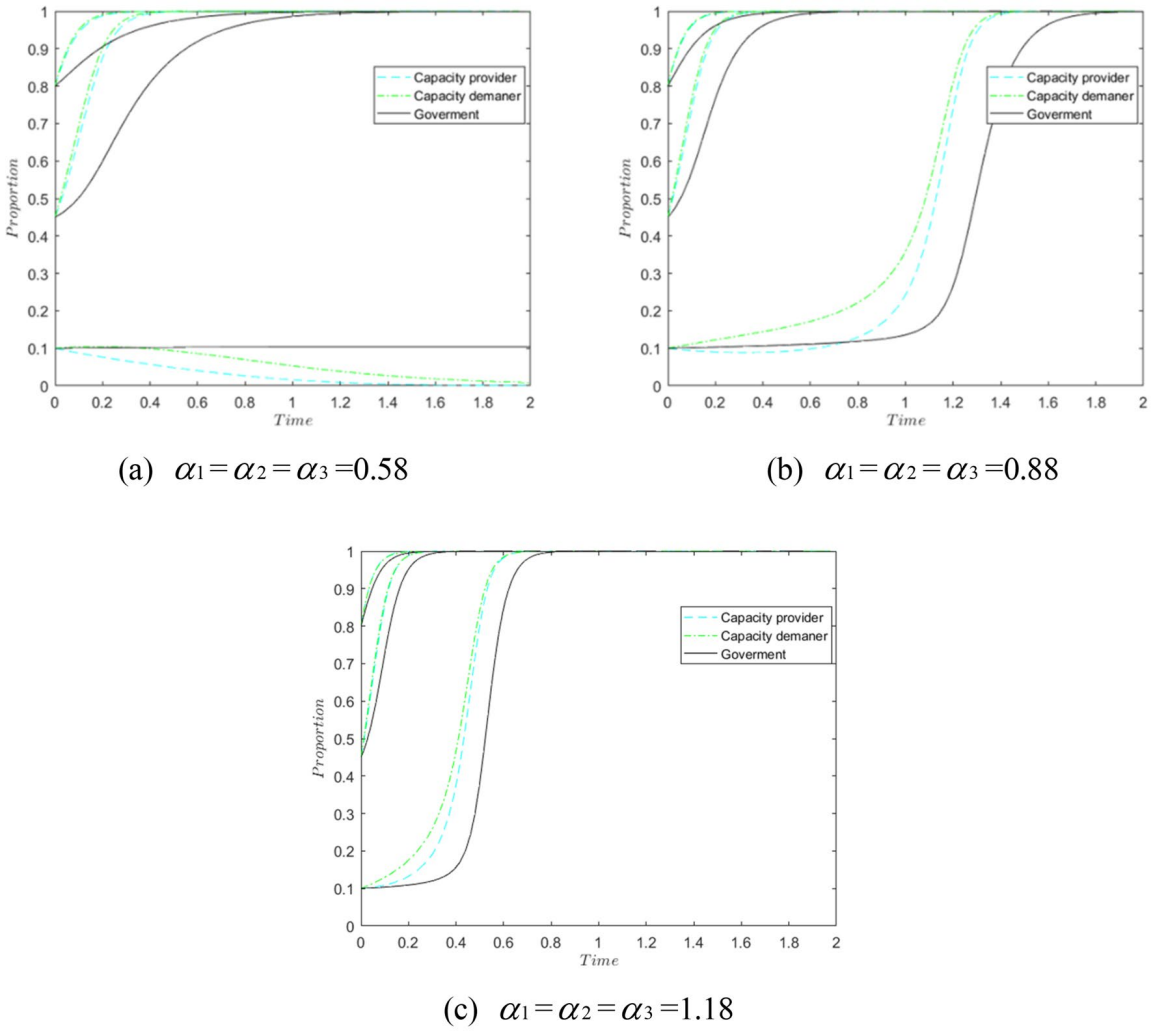
Impact of different risk sensitivity factors.

Influence of risk-sensitive coefficients. Change the risk sensitivity coefficients of the tripartite subjects so that $\alpha_{1}$, $\alpha_{2}$, $\alpha_{3}$ are 0.58, 0.88 and 1.18 respectively, and other values remain unchanged, and observe the simulation results as shown in Fig. 6.

As shown in Fig. 6a, the evolution of the system gradually tends to 0 when the initial probability is 0.1 and 1 when the initial probability is 0.45 and 0.8. With the increase of $\alpha_{1}$, $\alpha_2$ and $\alpha_{3}$, the evolution of the tripartite gradually accelerates to 1, and the capacity provider and the capacity demander are more inclined to take risks than the government. At the same time, the government, in order to gain good credibility, tends to choose the “sharing” or “receiving” policy when the capacity provider and the capacity demander cooperate. “Regulatory” policies. Therefore, the increase in the risk-sensitivity coefficient of the tripartite is conducive to the evolution of the system to the ideal state of 1, which is in the best interest.

Influence of loss aversion coefficients. Let $\lambda_{1}$, $\lambda_{2}$, $\lambda_{3}$ be 1.25, 2.25 and 3.25 respectively, and explore the change in the influence of the loss aversion coefficient on the tripartite.

**Figure 7:**
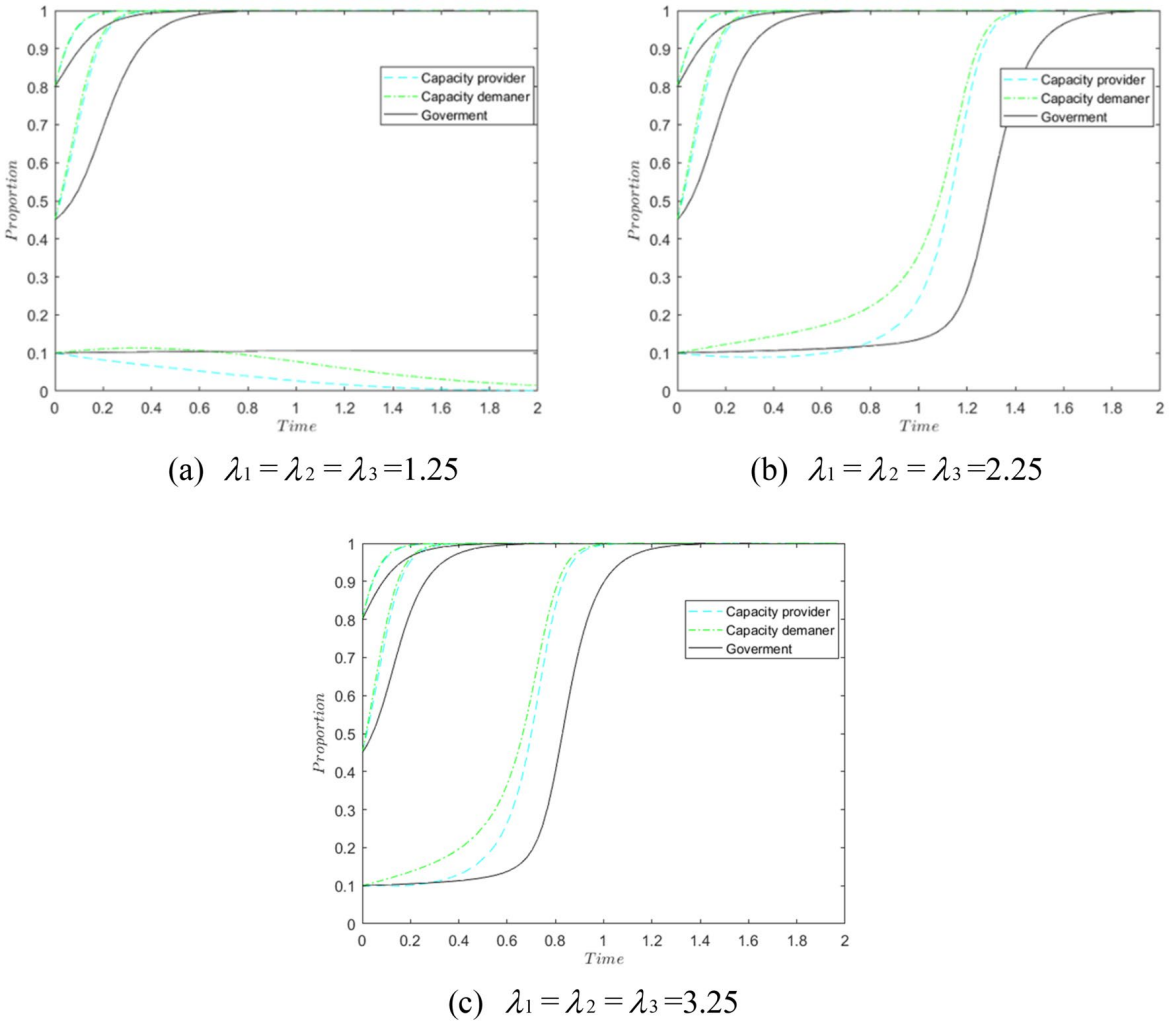
Impact of different loss aversion factors.

As shown in Fig. 7, when $\lambda_{1}$ = $\lambda_{2}$ = $\lambda_{3}$ = 1.25, the system gradually evolves to 0, and the capacity provider and capacity demander are more sensitive than the government; when $\lambda_{1}$ = $\lambda_{2}$ = $\lambda_{3}$ = 2.25 and 3.25, the system evolves to (1,1,1), and the higher the loss aversion coefficient, the faster the evolution; as the coefficient increases, the capacity provider and capacity demander increase their degree of loss aversion and are more inclined to As the coefficient increases, capacity providers and capacity demanders become more loss averse and are more likely to choose a “sharing” or “receiving” strategy. Therefore, the increase in the loss aversion coefficient is conducive to the evolution of the system to 1.

## Conclusion and management recommendations

### Conclusions and discussion

The uncertainty in the process of manufacturing capacity sharing leads to differences in decision-making among various subjects, and subjective perception affects the result of decision-making. Based on this, the article is constructed a tripartite game among manufacturing capacity providers, capacity demanders and the government based on prospect theory and evolutionary games, analyses the stability of the model using replicated dynamic equations, and simulates the values using MATLAB software.

The following conclusions are obtained: (1) Capacity sharing in the manufacturing industry is related to three parties, namely the capacity provider, the capacity demander and the government, and their strategies in the game process influence each other. When − ($\theta Q$ − $C_{2}$ + $R_{a}$ + $S_{2}$) < 0, − ($\theta Q$ − $C_{3}$ + $R_{b}$ + $S_{3}$) < 0, − ($R_{o}$ − $C_1$ − $S_{1}$ + $S_{o}$) < 0, the strategy combination finally evolves to the state of (1,1,1), i.e., under government regulation, the capacity provider chooses to “share” and the capacity demander chooses to “receive” The expected benefits outweigh the expected costs and losses. (2) Simulation of the values of risk sensitivity coefficient and loss avoidance coefficient with and without consideration respectively reveals that both will eventually evolve to (1,1,1), but the subject will have imperfect rationality in decision-making and will make decisions through various subjective judgments, so there will be small fluctuations in the evolution process. (3) The sensitivity of capacity providers and capacity demanders is higher than that of the government, and the increase in risk-sensitive coefficients will make capacity providers and capacity demanders perceive more benefits from capacity, and their choice to “share” or “receive” will gradually accelerate. At the same time, the government will also tend to choose a “regulatory” policy in order to gain good credibility. (4) Capacity providers and demanders are more loss-sensitive than the government, and the higher the loss aversion coefficient, the faster the evolution, and the increase in the loss aversion coefficient facilitates the evolution of the tripartite to 1.

According to the above conclusions, as the production capacity demand side and the production capacity provider are the main bodies in the manufacturing capacity sharing process, the production capacity provider and the production demand side are more sensitive to risk sensitivity and loss avoidance than the government. It may be because in the face of complex market environment, the supply and demand sides need to consider more, while the government, as a regulatory body, The main driving force is government intelligence, and the impact of decision-making on its reputation is mainly, so the sensitivity of the government to risk sensitivity and loss aversion is low. Under this result, the government can better play its functions under the condition of supervision, crack down on the unfair market environment, reduce sensitivity and promote the good development of the manufacturing industry.

### Recommendations

Based on the above conclusions, the article makes recommendations: (1) Government. The government should clarify its position, not only to regulate the industry, actively guide cooperation between enterprises and reduce their sunk costs, but also to actively carry out industry moral education and cultivate a sense of responsibility and morality among manufacturing enterprises; at the same time, the government can also set relevant laws and regulations to regulate the behavior of capacity providers and capacity demanders. (2) Capacity providers and capacity demanders should trust each other and work together to bring benefits to each other, thus promoting the good development of the manufacturing industry. (3) Platforms can be introduced, which match capacity between enterprises to ensure the full utilization of capacity.

### Innovation and shortcomings

This paper makes an evolutionary game analysis on the capacity provider, the demand side and the platform side of manufacturing capacity sharing, but ignores the perception difference of the main body in the decision-making process. Based on this, the paper introduces the prospect theory on the basis of the evolutionary game model, and finds that risk sensitivity and loss aversion have a great impact on participants through simulation. Based on the existing research, the results enrich the research of prospect theory in manufacturing capacity sharing, and a series of measures can be taken to reduce the cost and risk in the process of capacity sharing, so as to inject new impetus into the sustainable development of manufacturing industry.

The article was conducted under reasonable assumptions and did not analyze issues such as product completion time and product quality in the manufacturing capacity process, so product quality and delivery time factors can also be introduced for analysis; at the same time, platforms and blockchain technology can also be introduced for analysis in the future, which is also the direction of subsequent research.

### Supplementary Information

Supplementary Information.

## Data Availability

All data generated or analysed during this study are included in Supplementary Information files.
