# Supplementary material for: Research on the game of manufacturing capacity sharing based on prospect theory
Source: Sci Rep. 2023 Oct 23;13:18093. doi: 10.1038/s41598-023-45189-x (PMC10593761; doi:10.1038/s41598-023-45189-x)
Supplement: Supplementary file 1 — Supplementary Information. [file 41598_2023_45189_MOESM1_ESM.docx]

The analysed images were generated in MATLAB software and coded as follows:

**Figure 5-a.** Evolution in different contexts: No consideration of risk sensitivity and loss avoidance.

**Script 1-channeng：**

function dydt=channeng(t,y,θ,Q,c1,c2,c3,ro,ra,rb,so,s1,s2,s3)

dydt=zeros(3,1);

dydt(1)=y(1)*(1-y(1))*(y(2)*θ*Q-c2+ra+y(2)*s2);

dydt(2)=y(2)*(1-y(2))*(y(1)*θ*Q-c3+rb+y(1)*s3);

dydt(3)=y(3)*(1-y(3))*(y(1)*y(2)*(ro+c1-s1+so)+(y(1)+y(2))*(s1-c1));

end

**Script 2：**

θ=0.8,Q=20,c1=8,c2=4,c3=2,ro=13,ra=10,rb=8,so=10,s1=8,s2=5,s3=7;

for i=0.1:0.2:1

for j=0.1:0.2:1

for k=0.1:0.2:1

[t,y]=ode45(@(t,y) channeng(t,y,θ,Q,c1,c2,c3,ro,ra,rb,so,s1,s2,s3),[0 50],[i j k]);

figure(2)

grid on

plot3(y(:,1),y(:,2),y(:,3),'linewidth',1);

set(gca,'XTick',[0:0.2:1],'YTick',[0:0.2:1],'ZTick',[0:0.2:1])

hold on

axis([0 1 0 1 0 1])

view([-39 39])

end

end

end

xlabel('x','Rotation',0);

ylabel('y','Rotation',0);

zlabel('z','Rotation',360,'position',[1 1 1]);

title(' ','FontWeight','bold','position',[1 0 -0.13]);

**Figure 5-b.** Evolution in different contexts: Consider risk sensitivity and loss avoidance.

**Script 1-channeng：**

function dydt=channeng(t,y,θ,Q,c1,c2,c3,ro,ra,rb,so,s1,s2,s3,α,λ)

dydt=zeros(3,1);

dydt(1)=y(1)*(1-y(1))*(y(2)*θ*Q-c2+y(1)*ra^α+y(2)*(1-y(1))*λ*s2^α);

dydt(2)=y(2)*(1-y(2))*(y(1)*θ*Q-c3+y(1)*rb^α+y(1)*(1-y(2))*λ*s3^α);

dydt(3)=y(3)*(1-y(3))*(y(1)*y(2)*(y(3)*ro^α+c1-s1+(1-y(3))*λ*so^α))+(y(1)+y(2))*(s1-c1);

end

**Script 2：**

θ=0.8,Q=20,c1=8,c2=4,c3=2,ro=13,ra=10,rb=8,so=10,s1=8,s2=5,s3=7,α=0.88,λ=2.25;

for i=0.1:0.2:1

for j=0.1:0.2:1

for k=0.1:0.2:1

[t,y]=ode45(@(t,y) channeng(t,y,θ,Q,c1,c2,c3,ro,ra,rb,so,s1,s2,s3,α,λ),[0 50],[i j k]);

figure(2)

grid on

plot3(y(:,1),y(:,2),y(:,3),'linewidth',1);

set(gca,'XTick',[0:0.2:1],'YTick',[0:0.2:1],'ZTick',[0:0.2:1])

hold on

axis([0 1 0 1 0 1])

view([-39 39])

end

end

end

xlabel('x','Rotation',0);

ylabel('y','Rotation',0);

zlabel('z','Rotation',360,'position',[1 1 1]);

title(' ','FontWeight','bold','position',[1 0 -0.13]);

title(' ','FontWeight','bold','position',[1 0 -0.13]);

**Figure 6-a.** Impact of different risk sensitivity factors: ===0.58.

**Script 1-channeng：**

function dydt=channeng(t,y,θ,Q,c1,c2,c3,ro,ra,rb,so,s1,s2,s3,α,λ)

dydt=zeros(3,1);

dydt(1)=y(1)*(1-y(1))*(y(2)*θ*Q-c2+y(1)*ra^α+y(2)*(1-y(1))*λ*s2^α);

dydt(2)=y(2)*(1-y(2))*(y(1)*θ*Q-c3+y(1)*rb^α+y(1)*(1-y(2))*λ*s3^α);

dydt(3)=y(3)*(1-y(3))*(y(1)*y(2)*(y(3)*ro^α+c1-s1+(1-y(3))*λ*so^α))+(y(1)+y(2))*(s1-c1);

end

**Script 2：**

%%%%%%%%%%%%%%%%%%%%%%%%%%%%%%%%%%%%%%%%%%%%%%%%the 1rd

clc;

clear;

θ=0.8,Q=20,c1=8,c2=4,c3=2,ro=13,ra=10,rb=8,so=10,s1=8,s2=5,s3=7,α=0.58,λ=2.25;

set(0,'defaultfigurecolor','w')

% the 1st X,Y

[t,y]=ode45(@(t,y) channeng(t,y,θ,Q,c1,c2,c3,ro,ra,rb,so,s1,s2,s3,α,λ),[0 2],[0.1 0.1 0.1]);

figure(2)

h1=plot(t,y(:,1),'c--');

hold on

%%%%%%%%%%%%%%%%%%%%%%%%%%%%%%%%%%%%%%%%%%%%%%%%the 2rd

clc;

clear;

θ=0.8,Q=20,c1=8,c2=4,c3=2,ro=13,ra=10,rb=8,so=10,s1=8,s2=5,s3=7,α=0.58,λ=2.25;

set(0,'defaultfigurecolor','w')

% the 1st X,Y

[t,y]=ode45(@(t,y) channeng(t,y,θ,Q,c1,c2,c3,ro,ra,rb,so,s1,s2,s3,α,λ),[0 2],[0.45 0.45 0.45]);

figure(2)

h2=plot(t,y(:,1),'c--');

set(h2,'handlevisibility','off')

hold on

%%%%%%%%%%%%%%%%%%%%%%%%%%%%%%%%%%%%%%%%%%%%%%%%the 3rd

clc;

clear;

θ=0.8,Q=20,c1=8,c2=4,c3=2,ro=13,ra=10,rb=8,so=10,s1=8,s2=5,s3=7,α=0.58,λ=2.25;

set(0,'defaultfigurecolor','w')

% the 1st X,Y

[t,y]=ode45(@(t,y) channeng(t,y,θ,Q,c1,c2,c3,ro,ra,rb,so,s1,s2,s3,α,λ),[0 2],[0.8 0.8 0.8]);

figure(2)

h2=plot(t,y(:,1),'c--');

set(h2,'handlevisibility','off')

hold on

%%%%%%%%%%%%%%%%%%%%%%%%%%%%%%%%%%%%%%%%%%%%%%%%the 4rd

clc;

clear;

θ=0.8,Q=20,c1=8,c2=4,c3=2,ro=13,ra=10,rb=8,so=10,s1=8,s2=5,s3=7,α=0.58,λ=2.25;

set(0,'defaultfigurecolor','w')

% the 1st X,Y

[t,y]=ode45(@(t,y) channeng(t,y,θ,Q,c1,c2,c3,ro,ra,rb,so,s1,s2,s3,α,λ),[0 2],[0.1 0.1 0.1]);

figure(2)

h2=plot(t,y(:,2),'g-.');

hold on

%%%%%%%%%%%%%%%%%%%%%%%%%%%%%%%%%%%%%%%%%%%%%%%%the 5rd

clc;

clear;

θ=0.8,Q=20,c1=8,c2=4,c3=2,ro=13,ra=10,rb=8,so=10,s1=8,s2=5,s3=7,α=0.58,λ=2.25;

set(0,'defaultfigurecolor','w')

% the 1st X,Y

[t,y]=ode45(@(t,y) channeng(t,y,θ,Q,c1,c2,c3,ro,ra,rb,so,s1,s2,s3,α,λ),[0 2],[0.45 0.45 0.45]);

figure(2)

h2=plot(t,y(:,2),'g-.');

set(h2,'handlevisibility','off')

hold on

%%%%%%%%%%%%%%%%%%%%%%%%%%%%%%%%%%%%%%%%%%%%%%%%the 6rd

clc;

clear;

θ=0.8,Q=20,c1=8,c2=4,c3=2,ro=13,ra=10,rb=8,so=10,s1=8,s2=5,s3=7,α=0.58,λ=2.25;

set(0,'defaultfigurecolor','w')

% the 1st X,Y

[t,y]=ode45(@(t,y) channeng(t,y,θ,Q,c1,c2,c3,ro,ra,rb,so,s1,s2,s3,α,λ),[0 2],[0.8 0.8 0.8]);

figure(2)

h2=plot(t,y(:,2),'g-.');

set(h2,'handlevisibility','off')

hold on

%%%%%%%%%%%%%%%%%%%%%%%%%%%%%%%%%%%%%%%%%%%%%%%%the 7rd

clc;

clear;

θ=0.8,Q=20,c1=8,c2=4,c3=2,ro=13,ra=10,rb=8,so=10,s1=8,s2=5,s3=7,α=0.58,λ=2.25;

set(0,'defaultfigurecolor','w')

% the 1st X,Y

[t,y]=ode45(@(t,y) channeng(t,y,θ,Q,c1,c2,c3,ro,ra,rb,so,s1,s2,s3,α,λ),[0 2],[0.1 0.1 0.1]);

figure(2)

h2=plot(t,y(:,3),'k-');

hold on

%%%%%%%%%%%%%%%%%%%%%%%%%%%%%%%%%%%%%%%%%%%%%%%%the 8rd

clc;

clear;

θ=0.8,Q=20,c1=8,c2=4,c3=2,ro=13,ra=10,rb=8,so=10,s1=8,s2=5,s3=7,α=0.58,λ=2.25;

set(0,'defaultfigurecolor','w')

% the 1st X,Y

[t,y]=ode45(@(t,y) channeng(t,y,θ,Q,c1,c2,c3,ro,ra,rb,so,s1,s2,s3,α,λ),[0 2],[0.45 0.45 0.45]);

figure(2)

h2=plot(t,y(:,3),'k-');

set(h2,'handlevisibility','off')

hold on

%%%%%%%%%%%%%%%%%%%%%%%%%%%%%%%%%%%%%%%%%%%%%%%%the 9rd

clc;

clear;

θ=0.8,Q=20,c1=8,c2=4,c3=2,ro=13,ra=10,rb=8,so=10,s1=8,s2=5,s3=7,α=0.58,λ=2.25;

set(0,'defaultfigurecolor','w')

% the 1st X,Y

[t,y]=ode45(@(t,y) channeng(t,y,θ,Q,c1,c2,c3,ro,ra,rb,so,s1,s2,s3,α,λ),[0 2],[0.8 0.8 0.8]);

figure(2)

h2=plot(t,y(:,3),'k-');

set(h2,'handlevisibility','off')

hold on

axis( [0 2 0 1] )

xlabel('$Time$','interpreter','latex','Rotation',0);

ylabel('$Proportion$','interpreter','latex');

legend('Capacity provider','Capacity demaner','Goverment');

**Figure 6-b.** Impact of different risk sensitivity factors: ===0.88.

**Script 1-channeng：**

function dydt=channeng(t,y,θ,Q,c1,c2,c3,ro,ra,rb,so,s1,s2,s3,α,λ)

dydt=zeros(3,1);

dydt(1)=y(1)*(1-y(1))*(y(2)*θ*Q-c2+y(1)*ra^α+y(2)*(1-y(1))*λ*s2^α);

dydt(2)=y(2)*(1-y(2))*(y(1)*θ*Q-c3+y(1)*rb^α+y(1)*(1-y(2))*λ*s3^α);

dydt(3)=y(3)*(1-y(3))*(y(1)*y(2)*(y(3)*ro^α+c1-s1+(1-y(3))*λ*so^α))+(y(1)+y(2))*(s1-c1);

end

**Script 2：**

%%%%%%%%%%%%%%%%%%%%%%%%%%%%%%%%%%%%%%%%%%%%%%%%the 1rd

clc;

clear;

θ=0.8,Q=20,c1=8,c2=4,c3=2,ro=13,ra=10,rb=8,so=10,s1=8,s2=5,s3=7,α=0.88,λ=2.25;

set(0,'defaultfigurecolor','w')

% the 1st X,Y

[t,y]=ode45(@(t,y) channeng(t,y,θ,Q,c1,c2,c3,ro,ra,rb,so,s1,s2,s3,α,λ),[0 2],[0.1 0.1 0.1]);

figure(2)

h1=plot(t,y(:,1),'c--');

hold on

%%%%%%%%%%%%%%%%%%%%%%%%%%%%%%%%%%%%%%%%%%%%%%%%the 2rd

clc;

clear;

θ=0.8,Q=20,c1=8,c2=4,c3=2,ro=13,ra=10,rb=8,so=10,s1=8,s2=5,s3=7,α=0.88,λ=2.25;

set(0,'defaultfigurecolor','w')

% the 1st X,Y

[t,y]=ode45(@(t,y) channeng(t,y,θ,Q,c1,c2,c3,ro,ra,rb,so,s1,s2,s3,α,λ),[0 2],[0.45 0.45 0.45]);

figure(2)

h2=plot(t,y(:,1),'c--');

set(h2,'handlevisibility','off')

hold on

%%%%%%%%%%%%%%%%%%%%%%%%%%%%%%%%%%%%%%%%%%%%%%%%the 3rd

clc;

clear;

θ=0.8,Q=20,c1=8,c2=4,c3=2,ro=13,ra=10,rb=8,so=10,s1=8,s2=5,s3=7,α=0.88,λ=2.25;

set(0,'defaultfigurecolor','w')

% the 1st X,Y

[t,y]=ode45(@(t,y) channeng(t,y,θ,Q,c1,c2,c3,ro,ra,rb,so,s1,s2,s3,α,λ),[0 2],[0.8 0.8 0.8]);

figure(2)

h2=plot(t,y(:,1),'c--');

set(h2,'handlevisibility','off')

hold on

%%%%%%%%%%%%%%%%%%%%%%%%%%%%%%%%%%%%%%%%%%%%%%%%the 4rd

clc;

clear;

θ=0.8,Q=20,c1=8,c2=4,c3=2,ro=13,ra=10,rb=8,so=10,s1=8,s2=5,s3=7,α=0.88,λ=2.25;

set(0,'defaultfigurecolor','w')

% the 1st X,Y

[t,y]=ode45(@(t,y) channeng(t,y,θ,Q,c1,c2,c3,ro,ra,rb,so,s1,s2,s3,α,λ),[0 2],[0.1 0.1 0.1]);

figure(2)

h2=plot(t,y(:,2),'g-.');

hold on

%%%%%%%%%%%%%%%%%%%%%%%%%%%%%%%%%%%%%%%%%%%%%%%%the 5rd

clc;

clear;

θ=0.8,Q=20,c1=8,c2=4,c3=2,ro=13,ra=10,rb=8,so=10,s1=8,s2=5,s3=7,α=0.88,λ=2.25;

set(0,'defaultfigurecolor','w')

% the 1st X,Y

[t,y]=ode45(@(t,y) channeng(t,y,θ,Q,c1,c2,c3,ro,ra,rb,so,s1,s2,s3,α,λ),[0 2],[0.45 0.45 0.45]);

figure(2)

h2=plot(t,y(:,2),'g-.');

set(h2,'handlevisibility','off')

hold on

%%%%%%%%%%%%%%%%%%%%%%%%%%%%%%%%%%%%%%%%%%%%%%%%the 6rd

clc;

clear;

θ=0.8,Q=20,c1=8,c2=4,c3=2,ro=13,ra=10,rb=8,so=10,s1=8,s2=5,s3=7,α=0.88,λ=2.25;

set(0,'defaultfigurecolor','w')

% the 1st X,Y

[t,y]=ode45(@(t,y) channeng(t,y,θ,Q,c1,c2,c3,ro,ra,rb,so,s1,s2,s3,α,λ),[0 2],[0.8 0.8 0.8]);

figure(2)

h2=plot(t,y(:,2),'g-.');

set(h2,'handlevisibility','off')

hold on

%%%%%%%%%%%%%%%%%%%%%%%%%%%%%%%%%%%%%%%%%%%%%%%%the 7rd

clc;

clear;

θ=0.8,Q=20,c1=8,c2=4,c3=2,ro=13,ra=10,rb=8,so=10,s1=8,s2=5,s3=7,α=0.88,λ=2.25;

set(0,'defaultfigurecolor','w')

% the 1st X,Y

[t,y]=ode45(@(t,y) channeng(t,y,θ,Q,c1,c2,c3,ro,ra,rb,so,s1,s2,s3,α,λ),[0 2],[0.1 0.1 0.1]);

figure(2)

h2=plot(t,y(:,3),'k-');

hold on

%%%%%%%%%%%%%%%%%%%%%%%%%%%%%%%%%%%%%%%%%%%%%%%%the 8rd

clc;

clear;

θ=0.8,Q=20,c1=8,c2=4,c3=2,ro=13,ra=10,rb=8,so=10,s1=8,s2=5,s3=7,α=0.88,λ=2.25;

set(0,'defaultfigurecolor','w')

% the 1st X,Y

[t,y]=ode45(@(t,y) channeng(t,y,θ,Q,c1,c2,c3,ro,ra,rb,so,s1,s2,s3,α,λ),[0 2],[0.45 0.45 0.45]);

figure(2)

h2=plot(t,y(:,3),'k-');

set(h2,'handlevisibility','off')

hold on

%%%%%%%%%%%%%%%%%%%%%%%%%%%%%%%%%%%%%%%%%%%%%%%%the 9rd

clc;

clear;

θ=0.8,Q=20,c1=8,c2=4,c3=2,ro=13,ra=10,rb=8,so=10,s1=8,s2=5,s3=7,α=0.88,λ=2.25;

set(0,'defaultfigurecolor','w')

% the 1st X,Y

[t,y]=ode45(@(t,y) channeng(t,y,θ,Q,c1,c2,c3,ro,ra,rb,so,s1,s2,s3,α,λ),[0 2],[0.8 0.8 0.8]);

figure(2)

h2=plot(t,y(:,3),'k-');

set(h2,'handlevisibility','off')

hold on

axis( [0 2 0 1] )

xlabel('$Time$','interpreter','latex','Rotation',0);

ylabel('$Proportion$','interpreter','latex');

legend('Capacity provider','Capacity demaner','Goverment');

**Figure 6-c.** Impact of different risk sensitivity factors: ===1.18.

**Script 1-channeng：**

function dydt=channeng(t,y,θ,Q,c1,c2,c3,ro,ra,rb,so,s1,s2,s3,α,λ)

dydt=zeros(3,1);

dydt(1)=y(1)*(1-y(1))*(y(2)*θ*Q-c2+y(1)*ra^α+y(2)*(1-y(1))*λ*s2^α);

dydt(2)=y(2)*(1-y(2))*(y(1)*θ*Q-c3+y(1)*rb^α+y(1)*(1-y(2))*λ*s3^α);

dydt(3)=y(3)*(1-y(3))*(y(1)*y(2)*(y(3)*ro^α+c1-s1+(1-y(3))*λ*so^α))+(y(1)+y(2))*(s1-c1);

end

**Script 2：**

%%%%%%%%%%%%%%%%%%%%%%%%%%%%%%%%%%%%%%%%%%%%%%%%the 1rd

clc;

clear;

θ=0.8,Q=20,c1=8,c2=4,c3=2,ro=13,ra=10,rb=8,so=10,s1=8,s2=5,s3=7,α=1.18,λ=2.25;

set(0,'defaultfigurecolor','w')

% the 1st X,Y

[t,y]=ode45(@(t,y) channeng(t,y,θ,Q,c1,c2,c3,ro,ra,rb,so,s1,s2,s3,α,λ),[0 2],[0.1 0.1 0.1]);

figure(2)

h1=plot(t,y(:,1),'c--');

hold on

%%%%%%%%%%%%%%%%%%%%%%%%%%%%%%%%%%%%%%%%%%%%%%%%the 2rd

clc;

clear;

θ=0.8,Q=20,c1=8,c2=4,c3=2,ro=13,ra=10,rb=8,so=10,s1=8,s2=5,s3=7,α=1.18,λ=2.25;

set(0,'defaultfigurecolor','w')

% the 1st X,Y

[t,y]=ode45(@(t,y) channeng(t,y,θ,Q,c1,c2,c3,ro,ra,rb,so,s1,s2,s3,α,λ),[0 2],[0.45 0.45 0.45]);

figure(2)

h2=plot(t,y(:,1),'c--');

set(h2,'handlevisibility','off')

hold on

%%%%%%%%%%%%%%%%%%%%%%%%%%%%%%%%%%%%%%%%%%%%%%%%the 3rd

clc;

clear;

θ=0.8,Q=20,c1=8,c2=4,c3=2,ro=13,ra=10,rb=8,so=10,s1=8,s2=5,s3=7,α=1.18,λ=2.25;

set(0,'defaultfigurecolor','w')

% the 1st X,Y

[t,y]=ode45(@(t,y) channeng(t,y,θ,Q,c1,c2,c3,ro,ra,rb,so,s1,s2,s3,α,λ),[0 2],[0.8 0.8 0.8]);

figure(2)

h2=plot(t,y(:,1),'c--');

set(h2,'handlevisibility','off')

hold on

%%%%%%%%%%%%%%%%%%%%%%%%%%%%%%%%%%%%%%%%%%%%%%%%the 4rd

clc;

clear;

θ=0.8,Q=20,c1=8,c2=4,c3=2,ro=13,ra=10,rb=8,so=10,s1=8,s2=5,s3=7,α=1.18,λ=2.25;

set(0,'defaultfigurecolor','w')

% the 1st X,Y

[t,y]=ode45(@(t,y) channeng(t,y,θ,Q,c1,c2,c3,ro,ra,rb,so,s1,s2,s3,α,λ),[0 2],[0.1 0.1 0.1]);

figure(2)

h2=plot(t,y(:,2),'g-.');

hold on

%%%%%%%%%%%%%%%%%%%%%%%%%%%%%%%%%%%%%%%%%%%%%%%%the 5rd

clc;

clear;

θ=0.8,Q=20,c1=8,c2=4,c3=2,ro=13,ra=10,rb=8,so=10,s1=8,s2=5,s3=7,α=1.18,λ=2.25;

set(0,'defaultfigurecolor','w')

% the 1st X,Y

[t,y]=ode45(@(t,y) channeng(t,y,θ,Q,c1,c2,c3,ro,ra,rb,so,s1,s2,s3,α,λ),[0 2],[0.45 0.45 0.45]);

figure(2)

h2=plot(t,y(:,2),'g-.');

set(h2,'handlevisibility','off')

hold on

%%%%%%%%%%%%%%%%%%%%%%%%%%%%%%%%%%%%%%%%%%%%%%%%the 6rd

clc;

clear;

θ=0.8,Q=20,c1=8,c2=4,c3=2,ro=13,ra=10,rb=8,so=10,s1=8,s2=5,s3=7,α=1.18,λ=2.25;

set(0,'defaultfigurecolor','w')

% the 1st X,Y

[t,y]=ode45(@(t,y) channeng(t,y,θ,Q,c1,c2,c3,ro,ra,rb,so,s1,s2,s3,α,λ),[0 2],[0.8 0.8 0.8]);

figure(2)

h2=plot(t,y(:,2),'g-.');

set(h2,'handlevisibility','off')

hold on

%%%%%%%%%%%%%%%%%%%%%%%%%%%%%%%%%%%%%%%%%%%%%%%%the 7rd

clc;

clear;

θ=0.8,Q=20,c1=8,c2=4,c3=2,ro=13,ra=10,rb=8,so=10,s1=8,s2=5,s3=7,α=1.18,λ=2.25;

set(0,'defaultfigurecolor','w')

% the 1st X,Y

[t,y]=ode45(@(t,y) channeng(t,y,θ,Q,c1,c2,c3,ro,ra,rb,so,s1,s2,s3,α,λ),[0 2],[0.1 0.1 0.1]);

figure(2)

h2=plot(t,y(:,3),'k-');

hold on

%%%%%%%%%%%%%%%%%%%%%%%%%%%%%%%%%%%%%%%%%%%%%%%%the 8rd

clc;

clear;

θ=0.8,Q=20,c1=8,c2=4,c3=2,ro=13,ra=10,rb=8,so=10,s1=8,s2=5,s3=7,α=1.18,λ=2.25;

set(0,'defaultfigurecolor','w')

% the 1st X,Y

[t,y]=ode45(@(t,y) channeng(t,y,θ,Q,c1,c2,c3,ro,ra,rb,so,s1,s2,s3,α,λ),[0 2],[0.45 0.45 0.45]);

figure(2)

h2=plot(t,y(:,3),'k-');

set(h2,'handlevisibility','off')

hold on

%%%%%%%%%%%%%%%%%%%%%%%%%%%%%%%%%%%%%%%%%%%%%%%%the 9rd

clc;

clear;

θ=0.8,Q=20,c1=8,c2=4,c3=2,ro=13,ra=10,rb=8,so=10,s1=8,s2=5,s3=7,α=1.18,λ=2.25;

set(0,'defaultfigurecolor','w')

% the 1st X,Y

[t,y]=ode45(@(t,y) channeng(t,y,θ,Q,c1,c2,c3,ro,ra,rb,so,s1,s2,s3,α,λ),[0 2],[0.8 0.8 0.8]);

figure(2)

h2=plot(t,y(:,3),'k-');

set(h2,'handlevisibility','off')

hold on

axis( [0 2 0 1] )

xlabel('$Time$','interpreter','latex','Rotation',0);

ylabel('$Proportion$','interpreter','latex');

legend('Capacity provider','Capacity demaner','Goverment');

**Figure 7-a.** Impact of different loss aversion factors: ===1.25.

**Script 1-channeng：**

function dydt=channeng(t,y,θ,Q,c1,c2,c3,ro,ra,rb,so,s1,s2,s3,α,λ)

dydt=zeros(3,1);

dydt(1)=y(1)*(1-y(1))*(y(2)*θ*Q-c2+y(1)*ra^α+y(2)*(1-y(1))*λ*s2^α);

dydt(2)=y(2)*(1-y(2))*(y(1)*θ*Q-c3+y(1)*rb^α+y(1)*(1-y(2))*λ*s3^α);

dydt(3)=y(3)*(1-y(3))*(y(1)*y(2)*(y(3)*ro^α+c1-s1+(1-y(3))*λ*so^α))+(y(1)+y(2))*(s1-c1);

end

**Script 2：**

%%%%%%%%%%%%%%%%%%%%%%%%%%%%%%%%%%%%%%%%%%%%%%%%the 1rd

clc;

clear;

θ=0.8,Q=20,c1=8,c2=4,c3=2,ro=13,ra=10,rb=8,so=10,s1=8,s2=5,s3=7,α=0.88,λ=1.25;

set(0,'defaultfigurecolor','w')

% the 1st X,Y

[t,y]=ode45(@(t,y) channeng(t,y,θ,Q,c1,c2,c3,ro,ra,rb,so,s1,s2,s3,α,λ),[0 2],[0.1 0.1 0.1]);

figure(2)

h1=plot(t,y(:,1),'c--');

hold on

%%%%%%%%%%%%%%%%%%%%%%%%%%%%%%%%%%%%%%%%%%%%%%%%the 2rd

clc;

clear;

θ=0.8,Q=20,c1=8,c2=4,c3=2,ro=13,ra=10,rb=8,so=10,s1=8,s2=5,s3=7,α=0.88,λ=1.25;

set(0,'defaultfigurecolor','w')

% the 1st X,Y

[t,y]=ode45(@(t,y) channeng(t,y,θ,Q,c1,c2,c3,ro,ra,rb,so,s1,s2,s3,α,λ),[0 2],[0.45 0.45 0.45]);

figure(2)

h2=plot(t,y(:,1),'c--');

set(h2,'handlevisibility','off')

hold on

%%%%%%%%%%%%%%%%%%%%%%%%%%%%%%%%%%%%%%%%%%%%%%%%the 3rd

clc;

clear;

θ=0.8,Q=20,c1=8,c2=4,c3=2,ro=13,ra=10,rb=8,so=10,s1=8,s2=5,s3=7,α=0.88,λ=1.25;

set(0,'defaultfigurecolor','w')

% the 1st X,Y

[t,y]=ode45(@(t,y) channeng(t,y,θ,Q,c1,c2,c3,ro,ra,rb,so,s1,s2,s3,α,λ),[0 2],[0.8 0.8 0.8]);

figure(2)

h2=plot(t,y(:,1),'c--');

set(h2,'handlevisibility','off')

hold on

%%%%%%%%%%%%%%%%%%%%%%%%%%%%%%%%%%%%%%%%%%%%%%%%the 4rd

clc;

clear;

θ=0.8,Q=20,c1=8,c2=4,c3=2,ro=13,ra=10,rb=8,so=10,s1=8,s2=5,s3=7,α=0.88,λ=1.25;

set(0,'defaultfigurecolor','w')

% the 1st X,Y

[t,y]=ode45(@(t,y) channeng(t,y,θ,Q,c1,c2,c3,ro,ra,rb,so,s1,s2,s3,α,λ),[0 2],[0.1 0.1 0.1]);

figure(2)

h2=plot(t,y(:,2),'g-.');

hold on

%%%%%%%%%%%%%%%%%%%%%%%%%%%%%%%%%%%%%%%%%%%%%%%%the 5rd

clc;

clear;

θ=0.8,Q=20,c1=8,c2=4,c3=2,ro=13,ra=10,rb=8,so=10,s1=8,s2=5,s3=7,α=0.88,λ=1.25;

set(0,'defaultfigurecolor','w')

% the 1st X,Y

[t,y]=ode45(@(t,y) channeng(t,y,θ,Q,c1,c2,c3,ro,ra,rb,so,s1,s2,s3,α,λ),[0 2],[0.45 0.45 0.45]);

figure(2)

h2=plot(t,y(:,2),'g-.');

set(h2,'handlevisibility','off')

hold on

%%%%%%%%%%%%%%%%%%%%%%%%%%%%%%%%%%%%%%%%%%%%%%%%the 6rd

clc;

clear;

θ=0.8,Q=20,c1=8,c2=4,c3=2,ro=13,ra=10,rb=8,so=10,s1=8,s2=5,s3=7,α=0.88,λ=1.25;

set(0,'defaultfigurecolor','w')

% the 1st X,Y

[t,y]=ode45(@(t,y) channeng(t,y,θ,Q,c1,c2,c3,ro,ra,rb,so,s1,s2,s3,α,λ),[0 2],[0.8 0.8 0.8]);

figure(2)

h2=plot(t,y(:,2),'g-.');

set(h2,'handlevisibility','off')

hold on

%%%%%%%%%%%%%%%%%%%%%%%%%%%%%%%%%%%%%%%%%%%%%%%%the 7rd

clc;

clear;

θ=0.8,Q=20,c1=8,c2=4,c3=2,ro=13,ra=10,rb=8,so=10,s1=8,s2=5,s3=7,α=0.88,λ=1.25;

set(0,'defaultfigurecolor','w')

% the 1st X,Y

[t,y]=ode45(@(t,y) channeng(t,y,θ,Q,c1,c2,c3,ro,ra,rb,so,s1,s2,s3,α,λ),[0 2],[0.1 0.1 0.1]);

figure(2)

h2=plot(t,y(:,3),'k-');

hold on

%%%%%%%%%%%%%%%%%%%%%%%%%%%%%%%%%%%%%%%%%%%%%%%%the 8rd

clc;

clear;

θ=0.8,Q=20,c1=8,c2=4,c3=2,ro=13,ra=10,rb=8,so=10,s1=8,s2=5,s3=7,α=0.88,λ=1.25;

set(0,'defaultfigurecolor','w')

% the 1st X,Y

[t,y]=ode45(@(t,y) channeng(t,y,θ,Q,c1,c2,c3,ro,ra,rb,so,s1,s2,s3,α,λ),[0 2],[0.45 0.45 0.45]);

figure(2)

h2=plot(t,y(:,3),'k-');

set(h2,'handlevisibility','off')

hold on

%%%%%%%%%%%%%%%%%%%%%%%%%%%%%%%%%%%%%%%%%%%%%%%%the 9rd

clc;

clear;

θ=0.8,Q=20,c1=8,c2=4,c3=2,ro=13,ra=10,rb=8,so=10,s1=8,s2=5,s3=7,α=0.88,λ=1.25;

set(0,'defaultfigurecolor','w')

% the 1st X,Y

[t,y]=ode45(@(t,y) channeng(t,y,θ,Q,c1,c2,c3,ro,ra,rb,so,s1,s2,s3,α,λ),[0 2],[0.8 0.8 0.8]);

figure(2)

h2=plot(t,y(:,3),'k-');

set(h2,'handlevisibility','off')

hold on

axis( [0 2 0 1] )

xlabel('$Time$','interpreter','latex','Rotation',0);

ylabel('$Proportion$','interpreter','latex');

legend('Capacity provider','Capacity demaner','Goverment');

**Figure 7-b.** Impact of different loss aversion factors: ===2.25.

**Script 1-channeng：**

function dydt=channeng(t,y,θ,Q,c1,c2,c3,ro,ra,rb,so,s1,s2,s3,α,λ)

dydt=zeros(3,1);

dydt(1)=y(1)*(1-y(1))*(y(2)*θ*Q-c2+y(1)*ra^α+y(2)*(1-y(1))*λ*s2^α);

dydt(2)=y(2)*(1-y(2))*(y(1)*θ*Q-c3+y(1)*rb^α+y(1)*(1-y(2))*λ*s3^α);

dydt(3)=y(3)*(1-y(3))*(y(1)*y(2)*(y(3)*ro^α+c1-s1+(1-y(3))*λ*so^α))+(y(1)+y(2))*(s1-c1);

end

**Script 2：**

%%%%%%%%%%%%%%%%%%%%%%%%%%%%%%%%%%%%%%%%%%%%%%%%the 1rd

clc;

clear;

θ=0.8,Q=20,c1=8,c2=4,c3=2,ro=13,ra=10,rb=8,so=10,s1=8,s2=5,s3=7,α=0.88,λ=2.25;

set(0,'defaultfigurecolor','w')

% the 1st X,Y

[t,y]=ode45(@(t,y) channeng(t,y,θ,Q,c1,c2,c3,ro,ra,rb,so,s1,s2,s3,α,λ),[0 2],[0.1 0.1 0.1]);

figure(2)

h1=plot(t,y(:,1),'c--');

hold on

%%%%%%%%%%%%%%%%%%%%%%%%%%%%%%%%%%%%%%%%%%%%%%%%the 2rd

clc;

clear;

θ=0.8,Q=20,c1=8,c2=4,c3=2,ro=13,ra=10,rb=8,so=10,s1=8,s2=5,s3=7,α=0.88,λ=2.25;

set(0,'defaultfigurecolor','w')

% the 1st X,Y

[t,y]=ode45(@(t,y) channeng(t,y,θ,Q,c1,c2,c3,ro,ra,rb,so,s1,s2,s3,α,λ),[0 2],[0.45 0.45 0.45]);

figure(2)

h2=plot(t,y(:,1),'c--');

set(h2,'handlevisibility','off')

hold on

%%%%%%%%%%%%%%%%%%%%%%%%%%%%%%%%%%%%%%%%%%%%%%%%the 3rd

clc;

clear;

θ=0.8,Q=20,c1=8,c2=4,c3=2,ro=13,ra=10,rb=8,so=10,s1=8,s2=5,s3=7,α=0.88,λ=2.25;

set(0,'defaultfigurecolor','w')

% the 1st X,Y

[t,y]=ode45(@(t,y) channeng(t,y,θ,Q,c1,c2,c3,ro,ra,rb,so,s1,s2,s3,α,λ),[0 2],[0.8 0.8 0.8]);

figure(2)

h2=plot(t,y(:,1),'c--');

set(h2,'handlevisibility','off')

hold on

%%%%%%%%%%%%%%%%%%%%%%%%%%%%%%%%%%%%%%%%%%%%%%%%the 4rd

clc;

clear;

θ=0.8,Q=20,c1=8,c2=4,c3=2,ro=13,ra=10,rb=8,so=10,s1=8,s2=5,s3=7,α=0.88,λ=2.25;

set(0,'defaultfigurecolor','w')

% the 1st X,Y

[t,y]=ode45(@(t,y) channeng(t,y,θ,Q,c1,c2,c3,ro,ra,rb,so,s1,s2,s3,α,λ),[0 2],[0.1 0.1 0.1]);

figure(2)

h2=plot(t,y(:,2),'g-.');

hold on

%%%%%%%%%%%%%%%%%%%%%%%%%%%%%%%%%%%%%%%%%%%%%%%%the 5rd

clc;

clear;

θ=0.8,Q=20,c1=8,c2=4,c3=2,ro=13,ra=10,rb=8,so=10,s1=8,s2=5,s3=7,α=0.88,λ=2.25;

set(0,'defaultfigurecolor','w')

% the 1st X,Y

[t,y]=ode45(@(t,y) channeng(t,y,θ,Q,c1,c2,c3,ro,ra,rb,so,s1,s2,s3,α,λ),[0 2],[0.45 0.45 0.45]);

figure(2)

h2=plot(t,y(:,2),'g-.');

set(h2,'handlevisibility','off')

hold on

%%%%%%%%%%%%%%%%%%%%%%%%%%%%%%%%%%%%%%%%%%%%%%%%the 6rd

clc;

clear;

θ=0.8,Q=20,c1=8,c2=4,c3=2,ro=13,ra=10,rb=8,so=10,s1=8,s2=5,s3=7,α=0.88,λ=2.25;

set(0,'defaultfigurecolor','w')

% the 1st X,Y

[t,y]=ode45(@(t,y) channeng(t,y,θ,Q,c1,c2,c3,ro,ra,rb,so,s1,s2,s3,α,λ),[0 2],[0.8 0.8 0.8]);

figure(2)

h2=plot(t,y(:,2),'g-.');

set(h2,'handlevisibility','off')

hold on

%%%%%%%%%%%%%%%%%%%%%%%%%%%%%%%%%%%%%%%%%%%%%%%%the 7rd

clc;

clear;

θ=0.8,Q=20,c1=8,c2=4,c3=2,ro=13,ra=10,rb=8,so=10,s1=8,s2=5,s3=7,α=0.88,λ=2.25;

set(0,'defaultfigurecolor','w')

% the 1st X,Y

[t,y]=ode45(@(t,y) channeng(t,y,θ,Q,c1,c2,c3,ro,ra,rb,so,s1,s2,s3,α,λ),[0 2],[0.1 0.1 0.1]);

figure(2)

h2=plot(t,y(:,3),'k-');

hold on

%%%%%%%%%%%%%%%%%%%%%%%%%%%%%%%%%%%%%%%%%%%%%%%%the 8rd

clc;

clear;

θ=0.8,Q=20,c1=8,c2=4,c3=2,ro=13,ra=10,rb=8,so=10,s1=8,s2=5,s3=7,α=0.88,λ=2.25;

set(0,'defaultfigurecolor','w')

% the 1st X,Y

[t,y]=ode45(@(t,y) channeng(t,y,θ,Q,c1,c2,c3,ro,ra,rb,so,s1,s2,s3,α,λ),[0 2],[0.45 0.45 0.45]);

figure(2)

h2=plot(t,y(:,3),'k-');

set(h2,'handlevisibility','off')

hold on

%%%%%%%%%%%%%%%%%%%%%%%%%%%%%%%%%%%%%%%%%%%%%%%%the 9rd

clc;

clear;

θ=0.8,Q=20,c1=8,c2=4,c3=2,ro=13,ra=10,rb=8,so=10,s1=8,s2=5,s3=7,α=0.88,λ=2.25;

set(0,'defaultfigurecolor','w')

% the 1st X,Y

[t,y]=ode45(@(t,y) channeng(t,y,θ,Q,c1,c2,c3,ro,ra,rb,so,s1,s2,s3,α,λ),[0 2],[0.8 0.8 0.8]);

figure(2)

h2=plot(t,y(:,3),'k-');

set(h2,'handlevisibility','off')

hold on

axis( [0 2 0 1] )

xlabel('$Time$','interpreter','latex','Rotation',0);

ylabel('$Proportion$','interpreter','latex');

legend('Capacity provider','Capacity demaner','Goverment');

**Figure 7-c.** Impact of different loss aversion factors: ===3.25.

**Script 1-channeng：**

function dydt=channeng(t,y,θ,Q,c1,c2,c3,ro,ra,rb,so,s1,s2,s3,α,λ)

dydt=zeros(3,1);

dydt(1)=y(1)*(1-y(1))*(y(2)*θ*Q-c2+y(1)*ra^α+y(2)*(1-y(1))*λ*s2^α);

dydt(2)=y(2)*(1-y(2))*(y(1)*θ*Q-c3+y(1)*rb^α+y(1)*(1-y(2))*λ*s3^α);

dydt(3)=y(3)*(1-y(3))*(y(1)*y(2)*(y(3)*ro^α+c1-s1+(1-y(3))*λ*so^α))+(y(1)+y(2))*(s1-c1);

end

**Script 2：**

%%%%%%%%%%%%%%%%%%%%%%%%%%%%%%%%%%%%%%%%%%%%%%%%the 1rd

clc;

clear;

θ=0.8,Q=20,c1=8,c2=4,c3=2,ro=13,ra=10,rb=8,so=10,s1=8,s2=5,s3=7,α=0.88,λ=3.25;

set(0,'defaultfigurecolor','w')

% the 1st X,Y

[t,y]=ode45(@(t,y) channeng(t,y,θ,Q,c1,c2,c3,ro,ra,rb,so,s1,s2,s3,α,λ),[0 2],[0.1 0.1 0.1]);

figure(2)

h1=plot(t,y(:,1),'c--');

hold on

%%%%%%%%%%%%%%%%%%%%%%%%%%%%%%%%%%%%%%%%%%%%%%%%the 2rd

clc;

clear;

θ=0.8,Q=20,c1=8,c2=4,c3=2,ro=13,ra=10,rb=8,so=10,s1=8,s2=5,s3=7,α=0.88,λ=3.25;

set(0,'defaultfigurecolor','w')

% the 1st X,Y

[t,y]=ode45(@(t,y) channeng(t,y,θ,Q,c1,c2,c3,ro,ra,rb,so,s1,s2,s3,α,λ),[0 2],[0.45 0.45 0.45]);

figure(2)

h2=plot(t,y(:,1),'c--');

set(h2,'handlevisibility','off')

hold on

%%%%%%%%%%%%%%%%%%%%%%%%%%%%%%%%%%%%%%%%%%%%%%%%the 3rd

clc;

clear;

θ=0.8,Q=20,c1=8,c2=4,c3=2,ro=13,ra=10,rb=8,so=10,s1=8,s2=5,s3=7,α=0.88,λ=3.25;

set(0,'defaultfigurecolor','w')

% the 1st X,Y

[t,y]=ode45(@(t,y) channeng(t,y,θ,Q,c1,c2,c3,ro,ra,rb,so,s1,s2,s3,α,λ),[0 2],[0.8 0.8 0.8]);

figure(2)

h2=plot(t,y(:,1),'c--');

set(h2,'handlevisibility','off')

hold on

%%%%%%%%%%%%%%%%%%%%%%%%%%%%%%%%%%%%%%%%%%%%%%%%the 4rd

clc;

clear;

θ=0.8,Q=20,c1=8,c2=4,c3=2,ro=13,ra=10,rb=8,so=10,s1=8,s2=5,s3=7,α=0.88,λ=3.25;

set(0,'defaultfigurecolor','w')

% the 1st X,Y

[t,y]=ode45(@(t,y) channeng(t,y,θ,Q,c1,c2,c3,ro,ra,rb,so,s1,s2,s3,α,λ),[0 2],[0.1 0.1 0.1]);

figure(2)

h2=plot(t,y(:,2),'g-.');

hold on

%%%%%%%%%%%%%%%%%%%%%%%%%%%%%%%%%%%%%%%%%%%%%%%%the 5rd

clc;

clear;

θ=0.8,Q=20,c1=8,c2=4,c3=2,ro=13,ra=10,rb=8,so=10,s1=8,s2=5,s3=7,α=0.88,λ=3.25;

set(0,'defaultfigurecolor','w')

% the 1st X,Y

[t,y]=ode45(@(t,y) channeng(t,y,θ,Q,c1,c2,c3,ro,ra,rb,so,s1,s2,s3,α,λ),[0 2],[0.45 0.45 0.45]);

figure(2)

h2=plot(t,y(:,2),'g-.');

set(h2,'handlevisibility','off')

hold on

%%%%%%%%%%%%%%%%%%%%%%%%%%%%%%%%%%%%%%%%%%%%%%%%the 6rd

clc;

clear;

θ=0.8,Q=20,c1=8,c2=4,c3=2,ro=13,ra=10,rb=8,so=10,s1=8,s2=5,s3=7,α=0.88,λ=3.25;

set(0,'defaultfigurecolor','w')

% the 1st X,Y

[t,y]=ode45(@(t,y) channeng(t,y,θ,Q,c1,c2,c3,ro,ra,rb,so,s1,s2,s3,α,λ),[0 2],[0.8 0.8 0.8]);

figure(2)

h2=plot(t,y(:,2),'g-.');

set(h2,'handlevisibility','off')

hold on

%%%%%%%%%%%%%%%%%%%%%%%%%%%%%%%%%%%%%%%%%%%%%%%%the 7rd

clc;

clear;

θ=0.8,Q=20,c1=8,c2=4,c3=2,ro=13,ra=10,rb=8,so=10,s1=8,s2=5,s3=7,α=0.88,λ=3.25;

set(0,'defaultfigurecolor','w')

% the 1st X,Y

[t,y]=ode45(@(t,y) channeng(t,y,θ,Q,c1,c2,c3,ro,ra,rb,so,s1,s2,s3,α,λ),[0 2],[0.1 0.1 0.1]);

figure(2)

h2=plot(t,y(:,3),'k-');

hold on

%%%%%%%%%%%%%%%%%%%%%%%%%%%%%%%%%%%%%%%%%%%%%%%%the 8rd

clc;

clear;

θ=0.8,Q=20,c1=8,c2=4,c3=2,ro=13,ra=10,rb=8,so=10,s1=8,s2=5,s3=7,α=0.88,λ=3.25;

set(0,'defaultfigurecolor','w')

% the 1st X,Y

[t,y]=ode45(@(t,y) channeng(t,y,θ,Q,c1,c2,c3,ro,ra,rb,so,s1,s2,s3,α,λ),[0 2],[0.45 0.45 0.45]);

figure(2)

h2=plot(t,y(:,3),'k-');

set(h2,'handlevisibility','off')

hold on

%%%%%%%%%%%%%%%%%%%%%%%%%%%%%%%%%%%%%%%%%%%%%%%%the 9rd

clc;

clear;

θ=0.8,Q=20,c1=8,c2=4,c3=2,ro=13,ra=10,rb=8,so=10,s1=8,s2=5,s3=7,α=0.88,λ=3.25;

set(0,'defaultfigurecolor','w')

% the 1st X,Y

[t,y]=ode45(@(t,y) channeng(t,y,θ,Q,c1,c2,c3,ro,ra,rb,so,s1,s2,s3,α,λ),[0 2],[0.8 0.8 0.8]);

figure(2)

h2=plot(t,y(:,3),'k-');

set(h2,'handlevisibility','off')

hold on

axis( [0 2 0 1] )

xlabel('$Time$','interpreter','latex','Rotation',0);

ylabel('$Proportion$','interpreter','latex');

legend('Capacity provider','Capacity demaner','Goverment');
